# Epilepsy control with carbamazepine monotherapy from a genetic perspective

**DOI:** 10.1186/s40360-018-0261-y

**Published:** 2018-11-15

**Authors:** Shakir Ullah, Niaz Ali, Adnan Khan, Saad Ali, Haleema Rehna Nazish, Zia Uddin

**Affiliations:** 1 Department of Pharmacology Institute of Basic Medical Science, Khyber Medical University, Peshawar, Khyber Pakhtunkhwa Pakistan; 2 Department of Neurology, Govt. Lady Reading Hospital Peshawar, Peshawar, Khyber Pakhtunkhwa Pakistan

**Keywords:** Carbamazepine, High performance liquid chromatography, SNPs of MTHFR gene, Heterozygous variants, Plasma levels

## Abstract

**Background:**

Ethnicity variation is one of the main factors that may affect drug response in clinical practice. As MTHFR gene affects different transcriptome and proteome which affect the clinical response of drugs. Purpose of the current study was to observe possible variations in plasma levels of carbamazepine monotherapy and seizures’ control in Pakhtun population of Khyber Pakhtunkhwa (KP) in the context of MTHFR (C677T and A1298C) gene polymorphisms.

**Methods:**

Blood was collected from the epileptic patients treated with carbamazepine monotherapy for the first time following respective oral doses on its steady state concentration after 3 h of morning dose at 3^rd^ and 6^th^ month of the therapy. Plasma carbamazepine levels were determined using reverse phase high performance liquid chromatography after method validation. MTHFR (C677T, AA298C) gene was genotyped. Patients were followed on 3^rd^ and 6^th^ month of the therapy for monitoring of response to carbamazepine therapy.

**Results:**

Following for 3^rd^ and 6^th^ month of duration of carbamazepine therapy, poor seizure controlled patients were more likely noticed in heterozygous variants (677CT and 1298 AC) of MTHFR gene (*P* < 0.05). There was no significant (*P* > 0.05) difference in the dose and plasma level of carbamazepine among different genotypes of MTHFR (C677T and A1298C) gene. Similarly, the difference in dose and plasma level of carbamazepine was not significant (P > 0.05) in the responder and non-responder people with epilepsy.

**Conclusion:**

Our study suggests that heterozygous variants of MTHFR (C677T and A1298C) gene are associated with poor seizure control in Pakhtun population of KP despite the fact that plasma level of carbamazepine were found within the therapeutic range.

## Background

Carbamazepine (CBZ) is one of most commonly prescribed antiepileptic drugs (AEDs) for the treatment of partial and generalized tonic–clonic seizures [1].There is considerable interindividual variations in response to carbamazepine in clinical practice [2]. The variability in response to carbamazepine may be due to differences in pharmacokinetics, pharmacodynamics of drugs and ethnicity of individuals [3]. However, genetic variations are considered one of key factors that might be responsible for variable response to drug therapy in individualized patients [4]. Though some scientists believe that the determinants of variable response are still unknown [5]. MTHFR enzyme participates in remethylation of homocysteine to methionine and S-adenosylmethionine that helps in the methylation of DNA, synthesis of proteins and neurotransmitters [6]. MTHFR (C677T and A1298C) gene polymorphisms indirectly affect the metabolism of different drugs [7, 8] that may affect clinical response to carbamazepine therapy. Changes in MTHFR enzymatic activity may change the individual metabolome that could exert a pharmacogentic effect through one carbon metabolisms [7, 9–12]. Multiple studies have been carried out to know the pharmacogenomics effect of MTHFR gene polymorphisms on other drugs (for example methotrexate etc.) [13]. So far relations have been reported with MTHFR (C677T and A1298C) genotypes polymorphism and elevated levels of homocysteine due to vitamin B6, B12 and folic acid deficiency [13, 14]. More, we have recently observed that heterozygous variant (677CT and 1298 AC) of MTHFR gene frequently experienced low level of vitamin B6, which helps in synthesis of GABA, inhibitory neurotransmitters in carbamazepine treated patients. Hence, we postulated that the said heterozygous variant may be resistant to carbamazepine therapy as a consequence of vitamin B6 deficiency. Thus the current work is carried to correlate the clinical prognosis in heterozygous variant of MTHFR gene and GABRG2 gene, which are deficient in vitamin B6 as per our recently published work [14]. The pharmacogenomics effect on carbamazepine therapy remains largely unstudied in patients with epilepsies. The current study is focused on the possible pharmacogenomics effect of MTHFR (C677T and A1298C) gene polymorphisms on carbamazepine monotherapy and its subsequent control on seizures with carbamazepine therapy in Pakhtun population of Khyber Pakhtunkhwa.

## Methods

### Patients

The study was approved by the Ethics Board of the Khyber Medical University, Peshawar via approval no: DIR/KMU-EB/AC/000047, which complied with Helsinki’s declaration. Seventy-nine patients suffering from different types of partial and generalized seizures, without any co-morbidity, were purposively enrolled in the study at Outpatient Department of Neurology, Government Lady Reading Hospital, Peshawar, Pakistan from August 2014 to April 2016. Medical history, careful physical examinations and complete investigations were recorded on a standardized proforma by respective ward physicians. All patients signed written consents form while explaining the steps and the aims of the study in their local language (s).

### Sample size calculation, study design and protocols

**Fig. 1:**
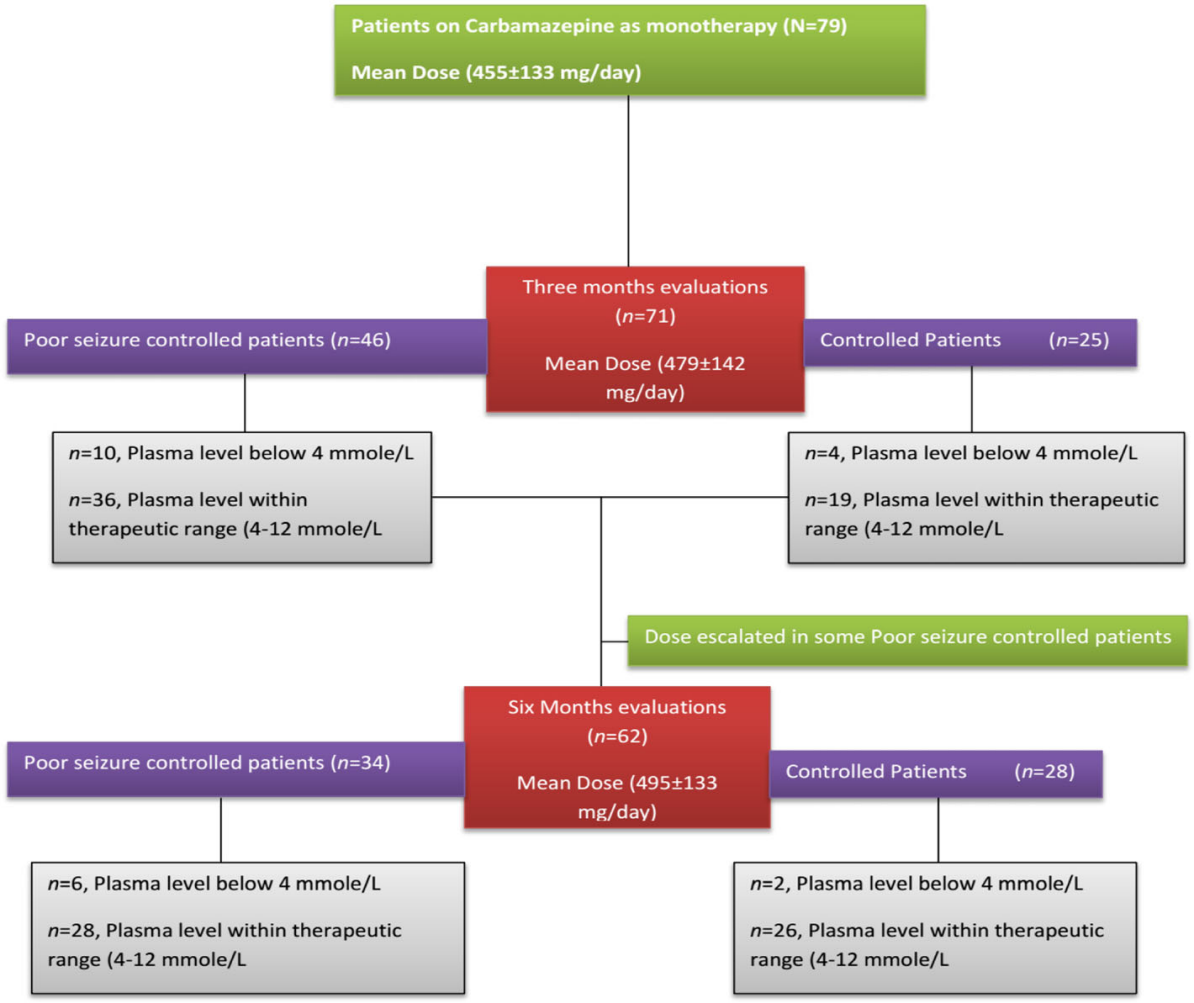
Schematic presentation of the study

Sample size (*n* = 62) were calculated at 95% CI, SD 4 and margin of error was 1. The alpha score was 0.025 and Z score was 1.95. We kept the Standard deviation (SD) and margin of error value low to authenticate the sample size. However, SD range from 3 to 11 and margin error 1–5 respectively. Mostly there are chances of drop out of enrolled patients during the study. So, keeping in view the chance of drop out from study we keep 20% drop out of the patients so we include 79 people with epilepsy at the base line. At the start of the therapy a baseline interviews were carried out to determine the types seizures and to assess the subsequent rational of the carbamazepine therapy. All the enrolled patients were treated with carbamazepine (Tegral 200 and 400 mg) monotherapy in a dose range of 200–800 mg/day on the basis of their seizures’ control keeping in view their types of epilepsies. Types of seizures were evaluated, based on clinical features, EEG and family history for epilepsy by concerned neurologist. Carbamazepine dose were prescribed on the basis severity of the seizures. Patients were asked to visit the Out-Patient Department of Neurology Department of Lady Reading Hospital, on monthly basis for monitoring of their clinical prognosis and to follow for adherence to carbamazepine therapy. The enrolled patients were evaluated on 3^rd^ and 6^th^ month of the carbamazepine therapy for clinical scoring for better epilepsy management and determining their mean plasma levels for carbamazepine. The doses of carbamazepine in patients with poor seizure controlled were escalated, based on patients’ prognosis by their respective ward/neuro physicians. The executing protocol of study is shown in Fig. 1.

### Inclusion criteria

Epileptic patients were enrolled in the study to whom carbamazepine as monotherapy for the first time was prescribed. Patients who were willing to participate in the study, after explaining the steps and the aim of the study in the context of local language, were included in the study upon consent and surrogate consent.

### Exclusion criteria

Patients were also excluded from the study that are not willing to participate in the study or was suffering from other co-morbidities and pregnant and feeding female patients.

### Blood sampling

Blood samples from each patient were taken for plasma therapeutic drug monitoring (TDM) at end of 3^rd^ month with correlation to mean dose administered on 3^rd^ and 6^th^ month of carbamazepine therapy after 3 h of morning dose. We focused on 3^rd^ and 6^th^ month as our project was also focusing on possible changes on other biochemical aspects, which may change in months. Blood samples from the targeted patients were taken in gel tubes following administration of morning dose of carbamazepine therapy assuring to be its on steady state concentrations as per its product monograph. The plasma was separated and stored for further analysis. For genotyping, blood samples were taken in EDTA tubes, stored on 8 °C till used for DNA extraction and subsequent genotyping.

### Genotyping of MTHFR (C677T and A1298C) gene

Genomic DNA was extracted using kit method (NucleoSpin® Blood, Germany). Briefly describing, the extraction of DNA was carried out through series of reactions, which included deproteination of blood (proteinase K), lysis of blood cell (lysis buffer), purification of DNA (washing buffer), and finally elution of DNA (through elution buffer) according to a standard protocols provided by its respective manufacturers. The extracted DNA was stored on − 20 °C for further analysis. MTHFR (C677T and A1298C) gene was amplified using gradient thermo cycler. After amplification, the PCR products were run on 2% agarose gel and assessed their size with 50 bp ladder. MTHFR (C677T) gene was amplified by using primers 5-TTTGAGGCTGACCTGAAGCACTTGAAGGAG-3 and primer 5-GAGTGGTAGCCCTGGATGGGAAAGATCCCG-3. The amplified product was digested using restriction enzyme *Hinf* I. Similarly, MTHFR (A1298C) gene was amplified by using primers 5-CTTTGGGGA GCTGAA GGACTACTAC-3 and 5-CACTTTGTG ACCATTCCGGTTTG-3.The resulted product was digested using restriction enzyme *Mbo*II. The digested products of each amplified products were run on 5% agarose gel and respective fragments sizes were assessed with 50 bp ladder [15]. The genotypes distributions were determined using the Hardy Weinberg Equilibrium (HWE) test to find the difference from world population. Adherence to HWE was tested using an online calculator <http://www.genes.org.uk/software/hardyweinberg.shtml>). A *P* value of greater than 0.05 indicated that the observed genotypes distributions were consistent with HWE assumptions.

### Analysis of plasma levels of carbamazepine

Carbamazepine dose-adjusted plasma concentrations were determined in epileptic patients on 3^rd^ and 6^th^ month of the therapy. Plasma from the centrifuged sample (0.2 ml) was added to 50 μl of 25% ammonium hydroxide (BDH Laboratory Supplies England) and 5 ml of chloroform HPLC grade (Scharlab S.L. Spain). The samples were mixed in a mechanical shaker for 20 min and centrifuged (PLC-05, Taiwan) at 3000 rpm for 10 min. After centrifugation the organic (chloroform) layer was transferred to another glass tube and evaporated by keeping the tube at 50 °C using water bath. The dry extract was reconstituted in 10 ml mobile phase. The extracted carbamazepine was analyzed using reverse phase high performance liquid chromatography (RP-HPLC) (HPLC LC-20AT Shimdzu Kyoto, Japan) coupled with UV detector SPD-20A/20AV (Shimdzu Kyoto Japan) with a slight modifications in method of Dordevic et al. (2009). Briefly describing, the mobile phase was a mixture of methanol (Chromosolve HPLC grade Cerritos, USA): demineralized water: glacial acetic acid 100% (Merck KGaA, Germany) at a ratio of 65:34:1 (*v*/v/v) with pH 5.6. The flow rate was 0.8 ml/min using C18 column (SEA 18, 5 μm 25 × 0.46 Mediterranean) with a load volume of 20 μl of samples. Detection of carbamazepine was performed in a UV range at λ _max_ 220 nm [16]. The validation parameter such % CV, precision, % accuracy and % recovery were tested and validated on intra and inter-day basis (unpublished data).

### Measurement of Seizure’s control

Seizures’ control was recorded in the form of reduction of frequencies seizures using standardized proforma. The numbers of seizures per week of the enrolled patients were recorded on baseline at the time of initiation of carbamazepine therapy (Tegral 200 and 400 mg) following evaluation for seizure control on 3^rd^ and 6^th^ month of the carbamazepine monotherapy. The patient compliance to the medication was checked by counting the pills remaining in the strip and feedback from already designed performance sheet for this purpose. Prognosis was determined as freedom from seizures after initiation of carbamazepine therapy on 3^rd^ and 6^th^ month of the therapy.

### Statistical analysis

Possible changes in dose and plasma levels of carbamazepine were compared after 3^rd^ and 6^th^ months of the carbamazepine therapy among different genotypes of MTHFR (C677T and A1298C) gene using one-way ANOVA followed by Tukey’s test. Changes in plasma level within responders and non-responders at 3^rd^ and 6^th^ month of the therapy were also compared using unpaired student “t” test with Welchs’ correction for high difference in SD. Differences in frequencies of seizures’ in carbamazepine non-responder people with epilepsy were also determined using unpaired student “t” test with Welchs’ correction for high difference in SD. The number of patients with epilepsy whose plasma level were below and within the therapeutic range was presented in percentage. Association of variant genotypes of MTHFR (C677T and A1298C) gene in people with epilepsy with poor responsive and responsive to carbamazepine therapy were determined using Chi^2^ square test with Yates’ correction at 95% CI, *P* ≤ 0.05 for to small sample size..

## Results

### Demographic and treatment plan

Patients’ demographic data and types of epilepsies (with respect to mean daily doses) are shown in Table 1. Generalized tonic clonic seizures were the most frequently (62%) reported type of epilepsies. The baseline initial mean dose was 455 ± 133 mg/day and titrated at mean dose of 24 ± 12.3 mg/day (479 ± 142 mg/day) and 16 ± 11.5 mg/day (495 ± 133 mg/day) on 3^rd^ month and 6^th^ month of the therapy (Table 2). Carbamazepine concentration-dose ratio (C/D) was 0.019 ± 0.007 at 3^rd^ month and 0.013 ± 0.006 at 6^th^ month of the therapy (Table 2).

**Table 1:** Demographic features of epileptic patient treated with carbamazepine monotherapy and seizures’ types

Variables	3^rd^ months of carbamazepine therapy (*n* = 71)	6^th^ months of carbamazepine therapy (*n* = 62)
Male, *n* (%)	34 (47.9)	28 (45.2)
Female, *n* (%)	37 (52.1)	34 (54.8)
Age (year) ± SD	17.7 ± 8.2	17.9 ± 8.6
Generalized Tonic Clonic Seizure, *n* (%)	46 (62.0)	44 (77.4)
Generalized Tonic Seizure, *n* (%)	4 (7.0)	1 (1.6)
Atonic Seizure, *n* (%)	2 (7.0)	2 (3.2)
Simple Partial Seizure, *n* (%)	5 (7.0)	3 (4.8)
Complex Partial Seizure, *n* (%)	4 (4.2)	3 (4.8)
Secondary Generalized Complex Seizure, *n* (%)	10 (7.0)	6 (9.7)

**Table 2:** Dose and Frequency of carbamazepine as monotherapy

Variables	Baseline (*n* = 79)	3^rd^ months (*n* = 71)	Dose Titration rate	6^th^ months (*n* = 62)	Dose Titration rate
Dose (mg/day)	455 ± 133	479 ± 142	24 ± 12.3	495 ± 133	16 ± 11.5
Frequency of dose					
OD, *n* (%)	30 (38.0)	12 (17.0)		8 (13.0)	
DIB, *n* (%)	35 (44.3)	39 (55.0)		30 (48.3)	
TID, *n* (%)	14 (17.7)	20 (28.0)		24 (38.7)	
Carbamazepine C/D Ratio	–	0.019 ± 0.007	–	0.013 ± 0.006	–

Once a day (*OD*); Two time a day (*BID*); three time a day (*TID*); concentration dose ratio (*C/D*)

### Distribution of MTHFR (C66T and A1298) gene genotypes

The distribution of genotypes of MTHFR (C677T) was found, according to the agreement of Hardy-Weinberg Equilibrium (HWE) because the *P* value was greater than 0.05 which is shown in Table 3. There was no significant (*P* > 0.05) difference in the distribution of genotypes of MTHFR (C677T) gene in different targeted study groups of patients using χ2 test (Table 3).

**Table 3:** Genotype distributions of MTHFR (C677T) gene polymorphisms in epileptic patients of Khyber Pakhtunkhwa (*n* = 79)

Patients treated with Carbamazepine as monotherapy	Genotype			
	CC, *n* (%)	CT, *n* (%)	TT, *n* (%)	HWE (*P* values)
MTHFR (C66T) (*n* = 79)	31 (0.40)	28 (0.35)	20 (0.25)	> 0.05
	AA	AC	CC	HWE (*P* values)
MTHFR (A1298C) (*n* = 79)	38 (0.48)	29 (0.37)	12 (0.15)	> 0.05

### Plasma levels of carbamazepine vs MTHFR (C677T and A1298C) gene polymorphisms

**Fig. 2:**
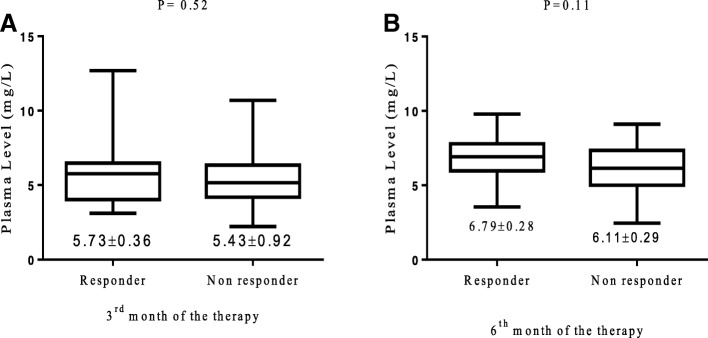
Carbamazepine plasma levels between the responsive and non-responsive groups at 3-month and 6-month. (**a**). 3^rd^ month of the therapy. (**b**). 6^th^ month of the therapy. Unpaired student t test was used to compare the plasma level of carbamazepine among responder and non-responder people with epilepsy

**Fig. 3:**
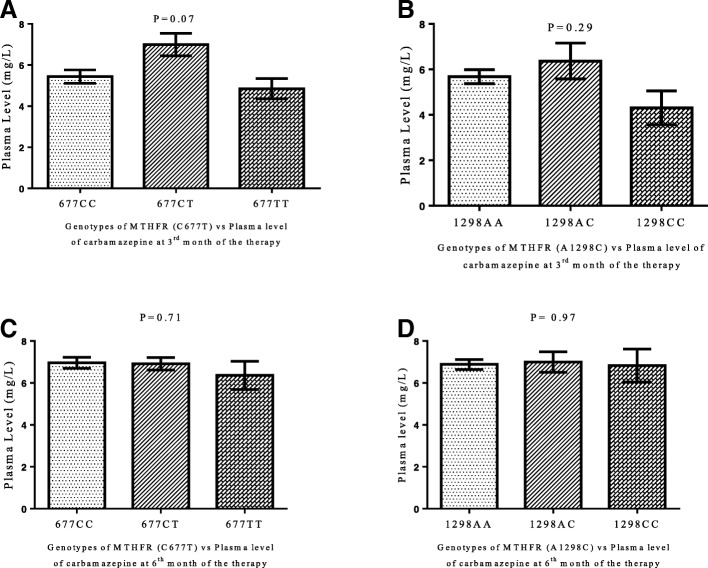
Carbamazepine plasma levels Vs genotypes at 3^rd^ month and 6^th^ month of the therapy. One Way ANOVA followed by Tukey’s test was used to compare the plasma level of carbamazepine among different genotypes of MTHFR (C677T and A1298C) gene polymorphisms

**Fig. 4:**
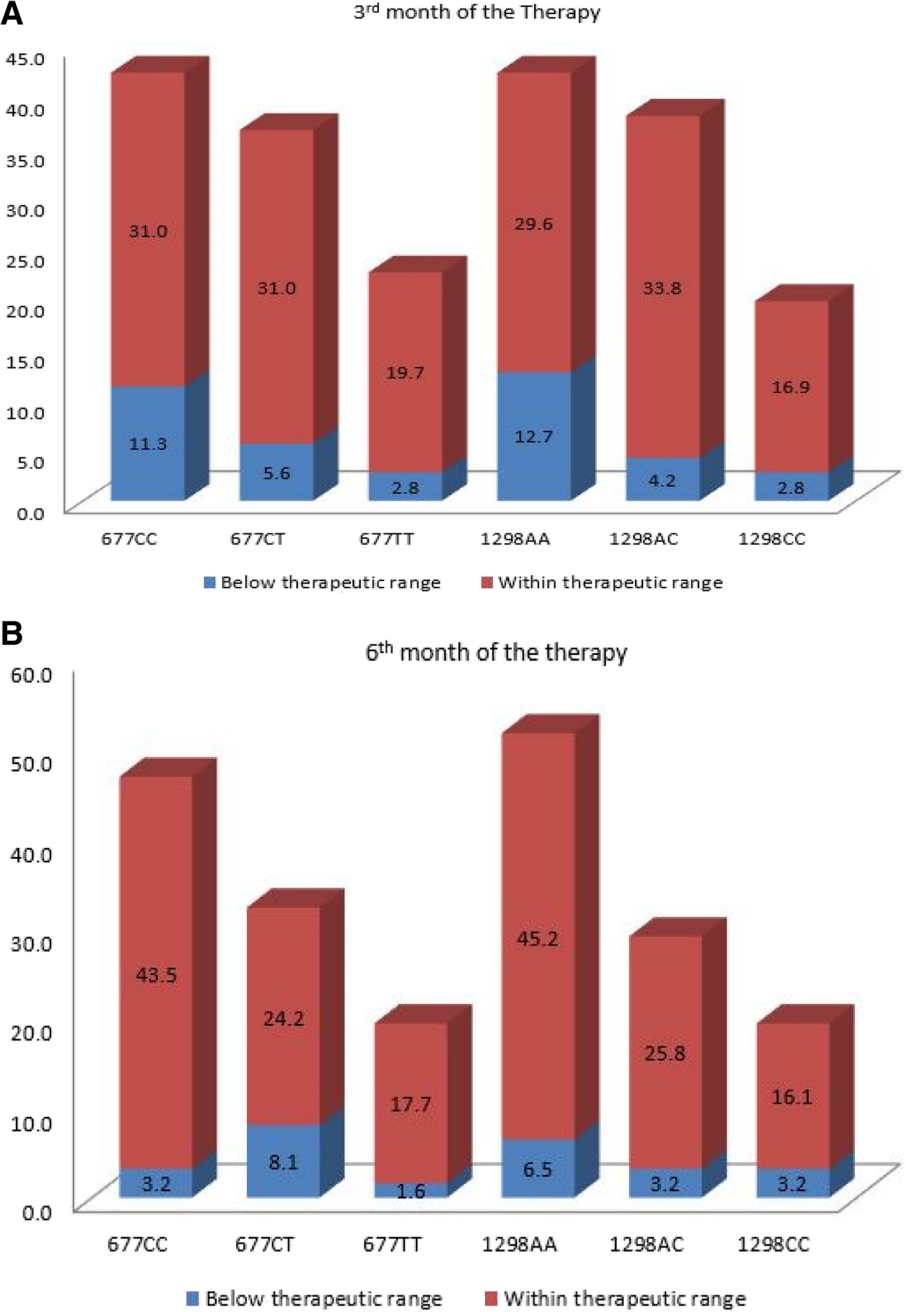
Proportion of people with epilepsy with low and within therapeutic range. (**a**). The number in block present the percentage (*n* = 71). (**b**). The number in block present the percentage (*n* = 62)

**Fig. 5:**
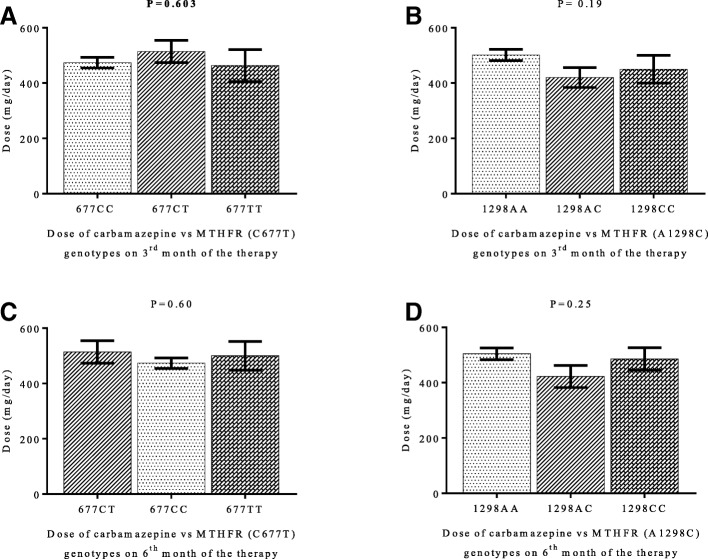
Dose of carbamazepine among different genotypes of MTHFR (C677T and A1298C) gene on 3^rd^ and 6^th^ month of the therapy. One-way ANOVA followed by Tukey’s test was used to compare the data

There was no variation in the plasma levels of carbamazepine in responder and non-responder patients on 3^rd^ and 6^th^ months of the therapy (Fig. 2a and b). Variations in patients’ plasma level of carbamazepine among different genotypes of MTHFR (C677T and A1298C) gene on 3^rd^ and 6^th^ month of the carbamazepine therapy were not significant (*P* > 0.05) with mean adjusted-dose of 479 ± 142 mg/day and 495 ± 133 mg/day (Fig. 3a, b, c and d). One way ANOV followed by Tukey’s test was used to evaluate the plasma level of carbamazepine among the genotype of MTHFR (C677T and A1298C) gene on 3^rd^ months and 6^th^ months of the therapy Fig. 3a, b, c and d). The proportion of people with epilepsy whose plasma level of carbamazepine were below and within the therapeutic range among different genotypes of MTHFR (C677T and A1298C) gene polymorphisms are shown in Fig. 4. It’s mean that response to carbamazepine was not affected the plasma level of carbamazepine in people with epilepsy. Furthermore, to evaluate the impact of dose of carbamazepine among different genotypes of MTHFR (C677T and A1298C) gene on the clinical response in people with epilepsy. It has been found that there was no significant (*P* > 0.05) difference in the dose of carbamazepine among different genotypes of MTHFR (C677T and A1298C) gene (Fig. 5a, b, c, d).

### MTHFR (C677T and A1298C) gene polymorphisms vs control of seizures on 3^rd^ month of the therapy

After 3^rd^ month of carbamazepine therapy, 46 patients were found resistant instead the plasma level of carbamazepine was in the therapeutic range. Following the people with epilepsy for three months, and seizure control are presented in Table 4. It has been observed that poor seizure controlled patients were more likely to have variants (677CT and 677TT) of MTHFR (C677T) gene than in patients whom seizures were controlled (χ^2^ = 6.75, *P* = 0.009 and χ2 = 1.5, *P* = 0.026) (Table 4). Similarly, poor seizure controlled patients were more likely to have variants (1298 AC, 1298CC) of MTHFR gene than its wild (1298AA) of MTHFR (A1298C) gene (χ^2^ = 7.2, *P* = 0.007 and 3.5, *P* = 0.05) (Table 4). Difference in frequency of seizures per week were significant (*P* < 0.05) in carbamazepine non responsive patients whose plasma level were below the therapeutic range compared to those carbamazepine non responsive patients whose plasma levels were within therapeutic range (Table 5).

**Table 4:** MTHFR (C677T and A1298C) gene polymorphisms with its relative frequencies in poor seizures controlled and seizure controlled patients at 3^rd^ month of carbamazepine monotherapy

*N* = 71	Carbamazepine non-responsive patients (*n* = 46)	Carbamazepine responsive patients (*n* = 25)
Variables	Total	Total
Number (%)	46 (65)	25 (35)
Genotypes		
677CC	14	16
677CT	21	4
677TT	11	5
1298AA	13	17
1298 AC	22	5
1298CC	11	3

MTHFR (C677T) Gene

Heterozygous (677CT) were more likely frequent in carbamazepine resistant patients compared to carbamazepine responsive patients in Pakhtun population of KP (χ^2^ = 6.75, *P* = 0.009)*) comparing total patients of carbamazepine resistant VS total patients who are responsive to carbamazepine therapy.

However, homozygous mutant (677TT) genotypes were less likely associated with carbamazepine therapy resistant patients as compared to carbamazepine responsive patients (χ2 = 1.5, *P* = 0.026)

MTHFR (A1298C) Gene

Heterozygous (1298 AC) genotypes were more likely frequent in carbamazepine resistant patients compared to carbamazepine responsive patients (χ^2^ = 7.2, *P* = 0.007**) comparing total patients of carbamazepine resistant VS total patients who are responsive to carbamazepine therapy.

However, homozygous mutant (1298CC) genotypes were less likely associated with carbamazepine therapy resistant patients as compared to carbamazepine responsive patients (χ^2^ = 3.5, *P* = 0.05).

**Table 5:** Frequency of seizures in non-responsive patients to carbamazepine monotherapy at 3^rd^ and 6^th^ month

Frequency of seizures	Baseline (*n* = 79)	At 3^rd^ month non-responsive (*n* = 46)		
		below range (*n* = 10)	within range (*n* = 36)	P Values
Frequency of seizures (numbers), Mean (SD)	8.3 ± 2.1	5.8 ± 1.9	3.9 ± 1.0	*t* = 5.6, *P* < 0.0001***
Frequency of seizures	Baseline (*n* = 79)	At 6^th^ month non-responsive (*n* = 34)		
		below range (*n* = 6)	within range (*n* = 28)	P Values
Frequency of seizures (numbers), Mean (SD)	8.3 ± 2.1	4.3 ± 0.4	3.5 ± 1.2	t = 5.6, *P* < 0.0001***

*** *P*<0.001 using 't' test with Welchs’ correction

### MTHFR (C677T and A1298C) gene polymorphisms vs control of seizures on 6^th^ month of the therapy

Following for 6^th^ months of carbamazepine therapy, it evident that 34 non-responder people with epilepsy were found, it is presented in Table 6. Poor seizure controlled patients were more likely to have variant (677CT and 677TT) genotypes than in controlled patients (χ^2^ = 7.5, *P* = 0.002 and 5.9, *P* = 0.01) (Table 6). Similarly, poor seizure controlled patients were more likely to have variant genotypes (1298 AC, 1298CC) than in controlled patients (χ^2^ = 7.03, *P* = 0.008 and 4.3, *P* = 0.03) (Table 6 self-explanatory). It has been found that heterozygous (677CT and 1298 AC) and homozygous mutant (677TT and 1298CC), who were resistant patients on 3^rd^ month of the therapy also remained resistant on 6^th^ month of the carbamazepine therapy. Difference in frequency of seizures per week were significant (*P* < 0.05) in carbamazepine non responsive patients whose plasma level were below the therapeutic range compared to carbamazepine non responsive patients whose plasma levels were within therapeutic range (Table 5).

**Table 6:** MTHFR (C677T and A1298C) gene polymorphisms with its relative frequencies in poor seizures controlled and seizure controlled patients at 6^th^ month of carbamazepine therapy

N = 62	Carbamazepine resistant patients (*n* = 34)	Carbamazepine responsive patients (*n* = 28)
Variables	Total	Total
Number (%)	34 (55)	28 (45)
Genotypes		
677CC	9	20
677CT	15	5
677TT	10	3
1298AA	11	21
1298 AC	14	4
1298CC	9	3

MTHFR (C677T) Gene

Heterozygous (677CT) genotypes were more likely frequent in carbamazepine resistant patients compared to carbamazepine responsive patients (χ^2^ = 7.5, *P* = 0.002**) comparing total patients of carbamazepine resistant *VS* total patients who are responsive to carbamazepine therapy. Similarly, Homozygous(677TT) genotypes were more likely frequent in carbamazepine therapy resistant patients as compared to carbamazepine responsive patients (χ^2^ = 5.9, *P* = 0.01*).

MTHFR (A1298C) Gene

Heterozygous (1298 AC) genotypes were more likely frequent in carbamazepine resistant patients compared to carbamazepine responsive patients (χ^2^ = 7.03, *P* = 0.008**) comparing total patients of carbamazepine resistant *VS* total patients who are responsive to carbamazepine therapy.

Similarly, homozygous (1298CC)genotypes were more likely frequent in carbamazepine therapy resistant patients as compared to carbamazepine responsive patients (χ^2^ = 4.3, *P* = 0.03*).

## Discussion

Therapeutic drug monitoring is commonly applied for drugs that have narrow therapeutic index. Here we followed patients for period of 6^th^ month to know about its possible shift in their plasma levels as well as its clinical prognosis. Our data showed a significant variation in the mean plasma levels of carbamazepine while comparing the above mentioned phases of study among different genotypes of MTHFR (C677T and A1298C) gene. Their plasma level remained within therapeutic range. Changes in plasma level of carbamazepine were only significant between wild genotype (677CC) of MTHFR gene on 3^rd^ month and 6^th^ month. Similarly, it was evident from post comparision test that changes were only significant in wild type (1298AA) of MTHFR gene on 3^rd^ month and 6^th^ month. This suggests that MTHFR (C677T and A1298C) gene polymorphisms did not directly affect the metabolism of carbamazepine in most patients and their plasma level remain within therapeutic range. It is known that many genes could influence the response to carbamazepine therapy. It is evident from our study that poor seizure controlled patients were more likely to have variants (677CT, 677TT and 1298 AC, 1298CC) of MTHFR gene. On the other hand, controlled patients have wild genotypes (677CC and 1298AA) of MTHFR gene. This could be due to the fact that vitamin B6 levels were low in variant (677CT and 677TT) genotypes of MTHFR (C677T) gene were more likely frequent in carbamazepine resistant patients as compared to carbamazepine responsive patients. Similarly, 1298 AC and 1298CC genotypes of MTHFR (C1298C) genotypes were more likely frequent in carbamazepine resistant patients as compared to carbamazepine responsive patients despite of their plasma level were in the recommended reference therapeutic level. Patients whose mean plasma levels were within therapeutic range (4–12 mg/L), their number of seizures and duration of seizures were less in carbamazepine resistant patients. These findings are consistent with the findings of Bauer et al. (2005), which says that clinical efficacy is related to steady state concentration within therapeutic range [17]. However, Ebid et al. (2007) reported that therapeutic steady state did not guarantee clinical effectiveness of a drug [18]. Hence, our observations can be supported by Schwahn et al. (2001) study that polymorphisms in the MTHFR gene lead to altered MTHFR activity may affect the individual metabolome either in shape of vitamin B6 deficiency that may be one cause for weakness of inhibitory neurons in CNS as vitamin B6 helps in synthesis of GABA. Thus it can, therefore, be a pharmacogenetic effect that still to be confirmed via knock out model in experimental animals (23). Similarly, Ulrich et al. (2002) imagines that indirect effect of MTHFR polymorphisms is commonly associated with variation in gene-specific or general DNA methylation, leading to differential gene expression. These differential expressions of genes affect different transcriptome and proteome, which may lead to drug variable response. Variable response to carbamazepine is multifactorial and many factors play an important role in pharmacogenomics of carbamazepine. But we are of opinion that variants (677CT, 677TT and 1298 AC, 1298CC) of MTHFR gene may have association with poor seizure control during carbamazepine therapy in epileptic Pakhtun patients of KP due to indirect metabolic effect through deficiency of vitamin B6 that helps in synthesis of inhibitory neurotransmitters. More, in these resistant patients, we found that vitamin B6 level was deficient in heterozygous variants of MTHFR gene [14]. The relation to plasma levels is not so much string as the genotypes are associated with clinical outcomes. However, the exact mechanistic approach is still out of the domain of this study as this could be multifaceted like attachment at receptors site of drugs or changes in monomers of GABA receptors that warrants further studies. The limitation of the study was that sample size was small and pharma scan or ADME chip analysis was import for proper exploration.

## Conclusion

Our study suggests that heterozygous variant genotypes of MTHFR (C677T and A1298C) gene are associated with poor seizure control in epilepsies treated with carbamazepine monotherapy despite their plasma levels were in therapeutic range pointing to possible pharmacodynamic interactions.

## Abbreviations

AEDs: Antiepileptic drugs
CBZ: Carbamazepine
HPLC: High Performance Liquid Chromatography
MTHFR: Methylene tetra hydrofolate reductase
